# Alleged Rape Perpetrated on a Female While under the Influence of Ether

**Published:** 1847-10

**Authors:** 


*Alleged Rape perpetrated on a Female while under the influence of Ether.*—That which had been suspected as a probable result, on the introduction of a new narcotizing agent, has, according to the *Gazette Médicale*, actually occurred in Paris. Last week a female went to a dentist to have a tooth extracted. He advised that it should be stopped; and, to avoid the pain of the operation, recommended his patient to inhale the vapour of ether. What passed while the female was under the influence of the vapour may be inferred from the following facts:—The young female was observed to leave the dentist's house about three hours after she had entered it, in a very disordered state. This attracted the attention of her employer, who could not account for her long absence. The injured party, notwithstanding the stupefying effects of the ether, retained some recollection of what had passed, and, from some words which fell from her, suspicion was immediately excited. She was examined by a physician, who reported that her person had been violated. The dentist has been arrested, and is about to be prosecuted for the offence.—*Ibid, from Gaz. Méd.*

